# Analytical and Clinical Sample Performance Characteristics of the Onclarity Assay for the Detection of Human Papillomavirus

**DOI:** 10.1128/JCM.02048-20

**Published:** 2020-12-17

**Authors:** Stephen Young, Laurence Vaughan, Karen Yanson, Karen Eckert, Aojun Li, James Harris, Aaron Ermel, James A. Williams, Mohammad Al-Ghoul, Catherine L. Cammarata, Stephanie N. Taylor, Ronald Luff, Charles K. Cooper, Barbara Van Der Pol

**Affiliations:** a Tricore Reference Laboratory, Albuquerque, New Mexico, USA; b Becton, Dickinson and Company, BD Life Sciences-Integrated Diagnostic Solutions, Sparks, Maryland, USA; c Indiana University School of Medicine, Indianapolis, Indiana, USA; d Center for Disease Detection, LLC, San Antonio, Texas, USA; e Louisiana State University Health Science Center, New Orleans, Louisiana, USA; f Quest Diagnostics, Teterboro, New Jersey, USA; g University of Alabama at Birmingham, Birmingham, Alabama, USA; Cepheid

**Keywords:** cervical cancer screening, atypical squamous cells of undetermined significance, human papillomavirus, genotype, cervical intraepithelial neoplasia, triage

## Abstract

The objective of this study was to determine the result reproducibility and performance of the BD Onclarity human papillomavirus (HPV) assay (Onclarity) on the BD Viper LT platform using both contrived and clinical specimens. Reproducibility was assessed in BD SurePath liquid-based cytology (LBC) medium (SurePath) using contrived panels (HPV genotype 16 [HPV16] positive, HPV18 positive, or HPV45 positive) or clinical specimens (HPV16, -18, -31, -33/58, -45, or -52 positive or HPV negative). In addition, specimens from 3,879 individuals from the Onclarity trial were aliquoted prior to or following cytology processing and tested for HPV. Finally, specimens were collected using either the Cervex-Brush or Cytobrush (or Cytobrush/spatula) for comparison of HPV results. Contrived specimens showed >95% concordance with the expected results, and pooled clinical specimens had standard deviations and coefficients of variation ranging from 0.87 to 1.86 and 2.9% to 5.6%, respectively. For precytology and postcytology aliquot analyses, specimens showed >98.0% overall agreement and mean differences in cycle threshold (*C_T_*) scores for HPV ranging from −0.07 to 0.31. Positivity rates were close between the Cervex-Brush and Cytobrush/spatula for all age groups tested. Onclarity results are reproducible and reliable, regardless of sample collection before or after cytology aliquoting. Onclarity performs well regardless of the method of specimen collection (Cervex-Brush or Cytobrush/spatula) for cervical cancer screening.

## Abstract (precis)

The objective of this study was to determine the result reproducibility and performance of the BD Onclarity human papillomavirus (HPV) assay (Onclarity) on the BD Viper LT platform using both contrived and clinical specimens. Reproducibility was assessed in BD SurePath liquid-based cytology (LBC) medium (SurePath) using contrived panels (HPV genotype 16 [HPV16] positive, HPV18 positive, or HPV45 positive) or clinical specimens (HPV16, -18, -31, -33/58, -45, or -52 positive or HPV negative). In addition, specimens from 3,879 individuals from the Onclarity trial were aliquoted prior to or following cytology processing and tested for HPV.

## INTRODUCTION

Over 95% of cervical cancer cases are caused by persistent infection with 13 to 14 human papillomavirus (HPV) genotypes (1–4), which can be clinically detected using assays targeting nucleic acid sequences in the HPV genome (5, 6). In the United States, 2019 ASCCP guidelines recommend a risk-based approach for cervical cancer screening and patient management following a positive screening result (7), and HPV testing is a key component of risk-based management in this context. An HPV-negative result provides an excellent negative predictive value to extend a woman’s screening interval to 5 years (8). In addition, extended genotyping has been shown to stratify risk in women with a negative cytology/positive HPV result to help determine those women at greatest risk for high-grade cervical disease or cancer, and nearly 30 countries in Europe utilize HPV testing in some capacity. Most programs currently involve HPV testing as part of cytology triage or cotesting. Countries such as Australia, The Netherlands, Italy, the United Kingdom, and Sweden have transitioned to HPV primary screening with cytology follow-up as necessary (9, 10).

The BD Onclarity HPV assay (“Onclarity”) (Becton, Dickinson and Company, BD Life Sciences-Integrated Diagnostic Solutions, Sparks, MD, USA) is clinically validated through U.S.- and non-U.S.-based studies for the detection of 14 high-risk HPV genotypes (11–13). Onclarity is FDA approved for reporting HPV genotype 16 (HPV16) and HPV18 during HPV primary screening (for women ≥25 years of age) and HPV16, -18, and -45 during ASCUS (atypical squamous cells of undetermined significance) triage (for women ≥21 years of age) and cotesting (for women ≥30 years of age); in addition, it is CE *in vitro* diagnostics (IVD) marked for the detection of 14 high-risk HPV genotypes (14–16). In addition, Onclarity is FDA approved for reporting individual genotype results for HPV16, -18, -31, -45, -51, and -52 and grouped results for HPV33/58, -35/29/68, and -56/59/66 in order to facilitate risk-based screening for cervical cancer and precancer.

The Onclarity assay is performed on samples obtained from liquid-based cytology (LBC) specimens collected using a Cervex-Brush or Cytobrush (or Cytobrush/spatula) device. However, the order of aliquoting for the Onclarity assay can vary based on the respective screening strategy employed by each laboratory. For example, sites utilizing HPV primary screening with cytology triage will perform HPV testing from an initial LBC aliquot (precytology) and use the remaining vial/specimen for cytology. Conversely, sites employing a screening program with primary cytology testing and HPV triage testing will perform cytology testing first, followed by HPV testing from the specimen after cytology processing (postcytology aliquot). Therefore, it is important to establish that the performance of the HPV assay is unaffected by the order in which the aliquot is taken.

Contrived specimens and pooled clinical specimens were utilized to test reproducibility within Onclarity assay runs and between Onclarity assay runs, study sites, operators, reagent lots, and days of operation. In addition, data from pre- and postcytology aliquot specimens were analyzed to determine whether the Onclarity assay performance is impacted by the order in which the sample is aliquoted (i.e., before or after cytology). Finally, from BD SurePath (“SurePath”) (Becton, Dickinson and Company, BD Life Sciences-Integrated Diagnostic Solutions, Sparks, MD, USA) vials obtained during the Onclarity trial, Onclarity assay results were compared in specimens obtained using two different collection devices in order to determine whether performance results are affected based on the method of sampling for endocervical specimens.

## MATERIALS AND METHODS

### Clinical trial population.

Women ≥21 years of age (women >65 years of age were included if they met U.S. Preventive Services Task Force (USPSTF) screening recommendations) were invited to join the Onclarity trial between 2013 and 2015. Initially, 33,858 subjects (across 31 collection sites) were enrolled; the trial population, criteria for inclusion/exclusion, and procedures involving LBC collection, cytology testing, colposcopy/biopsy procedures, and histology examination/diagnosis were described previously (14). By cytology, 30,489 women characterized as negative for intraepithelial lesions or malignancies (NILM) cytology, 1,960 women identified with atypical squamous cells of undetermined significance (ASCUS) cytology, and 1,122 women identified with >ASCUS cytology (where ASCUS stands for atypical squamous cells of undetermined significance) were included in the baseline data from the Onclarity trial. The study was approved by institutional review boards at each study site, and written informed consent was obtained prior to any trial-related procedures; this study was conducted according to the principles set forth by the Declaration of Helsinki and good clinical practice, and this report was prepared according to STARD (Standards for Reporting of Diagnostic Accuracy) guidelines for reporting diagnostic accuracy.

### Preparation for clinical reproducibility, pre- and postcytology aliquots, and collection device experiments.

For reproducibility testing, contrived panel members were prepared using SiHa, HeLa, and MS751 transformed cell lines that express HPV16, -18, and -45, respectively. Aliquots from each cell panel preparation were added to an HPV-negative SurePath clinical matrix to yield high-negative specimens (*C*_5_ [specimens called positive approximately 5% and negative 95% of the time]), low-positive specimens (*C*_95_ [specimens called positive approximately 95% and negative 5% of the time]), and moderate-positive specimens (3× *C*_95_ [specimens approximately three times above the *C*_95_ level and expected to be positive 100% of the time]). These determinations were made based on the assay cycle threshold (*C_T_*) values relative to the clinical cutoff point (*C*_95_) associated with the assay. Pooled clinical specimens positive for HPV16, -18, -45, -31, -33/58, or -52 were diluted (with the HPV-negative clinical specimen matrix) to a detection level close to the *C*_95_ (the clinical cutoff). Negative panel members were created by pooling high-risk-HPV-negative clinical specimens. All panel members were stored at −20°C prior to Onclarity assay testing. Standard deviations and coefficients of variability for PCR mean cycle times within a run, between runs, between operators, between sites, between reagent lots, and between days (see Fig. S1 in the supplemental material) were all factors used as outcome measures of reproducibility.

During the Onclarity trial, endocervical specimens were collected using a Rovers Cervex-Brush (Rovers Medical Devices, The Netherlands) or a Cytobrush Plus GT and a Pap Perfect plastic spatula (“Cytobrush/spatula”) (Cooper Surgical, Inc., Trumbull, CT, USA) and stored/transported in SurePath LBC specimen vials. Clinical specimens were processed (as described below) and utilized for HPV testing via the Onclarity assay on the BD Viper LT system (“Viper LT”) (Becton, Dickinson and Company, BD Life Sciences-Integrated Diagnostic Solutions, Sparks, MD, USA).

For precytology and postcytology specimens, central laboratory personnel vortexed the SurePath LBC specimen and manually aliquoted 0.5 ml of the specimen into an HPV LBC diluent tube (precytology aliquot). Aliquoting from SurePath LBC specimen vials to diluent tubes was performed in the same order as the specimen vials were received. Following the removal of the 0.5-ml precytology aliquot, 8.0 ml of the specimen was removed from the SurePath LBC vial, and a cytology slide was processed (using the BD PrepMate/PrepStain system; Becton, Dickinson and Company, BD Life Sciences-Integrated Diagnostic Solutions, Sparks, MD, USA) according to the manufacturer’s instructions. A final 0.5-ml aliquot from residual fluid in the SurePath LBC vial was manually transferred into a second HPV LBC diluent tube (postcytology aliquot). Thus, pre- and postcytology aliquot diluent tubes were obtained from the same SurePath LBC specimen vials; both diluent tubes were sent to one of four laboratories that ran Viper LT testing (Fig. S2). There was a minimal delay for the postcytology aliquot specimens while cytology slides were prepared; this was within the validated room-temperature storage time. Overall, 3,879 SurePath vials were utilized in the study to provide pre- and postcytology aliquot pairs for HPV testing and analysis.

### Sample processing for HPV testing.

The details for HPV testing with the Onclarity assay on the Viper LT system using LBC specimens were described previously (16, 17). Briefly, Onclarity uses three processing steps: (i) an aliquoted, collected specimen matrix in SurePath medium is vortexed and prewarmed; (ii) the nucleic acids are extracted using BD Fox extraction (Becton, Dickinson and Company, BD Life Sciences-Integrated Diagnostic Solutions, Sparks, MD, USA) that involves automated matrix homogenization, cell lysis, binding, and elution of DNA; and (iii) real-time PCR amplification of both HPV E6/E7 and human β-globin (HBB) target DNA sequences is performed on the Viper LT system. TaqMan DNA probes (Thermo Fisher, Pittsburgh, PA, USA) include a fluorescent dye at the 5′ end and a quenching molecule at the 3′ end of the oligonucleotide. Three individual PCR tubes (G1, G2, and G3) collectively detect 14 high-risk HPV genotypes (6 individual genotypes, 16, 18, 31, 45, 51, and 52, and three groups containing 8 genotypes, 33/58, 35/39/68, and 56/59/66). The human beta globin gene served as the internal control for each PCR across all three PCR tubes.

### Data collection and analysis.

For reproducibility testing, three test sites analyzed panels, testing one panel in duplicate (once per operator) daily, for 9 days. Three different reagent lots were utilized: one lot per 3 days of testing. Panel members were randomized, and technical staff were blind to genotypes in each panel member. A total of 162 results (54 per testing site) were expected for each panel member. Percent agreement (with the accompanying lower and upper 95% confidence intervals) analyses were performed for high-negative, low-positive, and moderate-positive contrived specimens. The acceptance criterion for HPV assay performance during testing of panel members was predetermined: for low-positive specimens, it was 94%, and for moderate-positive specimens, it was 98% (Table S1). For clinical specimen analysis, specific mean *C_T_* scores (between 34.2 and 38.3 for HPV16 and between 29.6 and 34.2 for the other 13 genotypes) were required to ensure that genotypes were being detected in proximity to the clinical cutoff. The limit of detection around the clinical cutoffs for HPV16 (*C_T_* value of 38.3) is around 1,500 viral genome copies/ml of undiluted SurePath medium; for the other 13 genotypes (*C_T_* value of 34.2), it ranges from 3,000 to 10,000 viral genome copies/ml. Additional information regarding this issue is available in the product’s information-for-use document (18).

For pre- versus postcytology aliquot comparison, positive, negative, and overall agreements were determined using the precytology aliquot result to define positive and negative. Mean (with lower and upper 95% confidence intervals) pre- and postcytology aliquot *C_T_* scores, including mean differences between the two, were calculated, and statistical comparison was performed using a paired *t* test. Linear regression was performed for high-risk HPV genotype detection between pre- and postcytology aliquot specimens.

Data for comparison of collection devices were generated at four testing sites in the United States from a precytology aliquot. HBB *C_T_* scores, HPV *C_T_* scores, and high-risk HPV positivity rates were analyzed in three intended-use populations (ASCUS, ≥21 years of age; NILM, ≥30 years of age; and primary screening, ≥25 years of age) and different age groups (21 to 24, 25 to 29, 30 to 39, 40 to 49, and ≥50 years of age). The mean HBB *C_T_* score was calculated by averaging each specimen’s three internal control *C_T_* score results. The HPV *C_T_* score was calculated by selecting the strongest *C_T_* score from nine channels, excluding subjects without an HPV *C_T_* score result. The HBB and HPV *C_T_* scores were compared using a two-sample *t* test. The *P* value that was determined using the Satterthwaite approximation for degrees of freedom was reported. To test whether HPV positivity rates were different between the two collection devices, Fisher’s two-sided exact test was performed.

## RESULTS

### Onclarity assay reproducibility.

**FIG 1:**
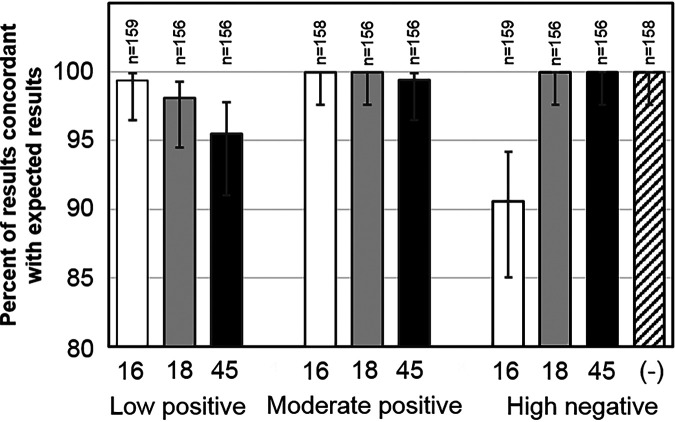
Contrived specimens positive for HPV16, -18, and -45 were tested with the Onclarity assay. The contrived specimens were prepared at concentrations categorized as “low positive,” “moderate positive,” and “high negative”; all three are characterized relative to the clinical cutoff. Results from the Onclarity assay for each of the three contrived sample groups were compared to the expected results. An HPV-negative group was included with the high-negative contrived sample run.

For reproducibility testing, contrived specimens were created using cells expressing HPV16 (SiHa), HPV18 (HeLa), and HPV45 (MS751) to spike an HPV-negative clinical specimen matrix at prespecified low- and moderate-positive concentrations. As shown in Fig. 1 (see also Table S2 in the supplemental material), the Onclarity assay reported results for HPV16, -18, and -45 that were all above 95% agreement within the low-positive panels and near 100% for the moderate-positive panels (both compared to the expected results). For the pooled HPV high-negative clinical panels, 91.6% of the samples were negative for HPV16, whereas 100% of the HPV18 and HPV45 samples returned a correct result of negative. For pooled clinical specimens positive for HPV16, -18, -45, -31, -33/58, or -52, the reproducibility for the mean *C_T_* score met the acceptance criteria; the overall standard deviations and percent coefficients of variation ranged from 0.87 to 1.86 and 2.9% to 5.6%, respectively, with the greatest variation being observed within replicates on the same instrument run (Table 1). HPV-negative samples (HPV-negative clinical matrix or HPV-negative cell line suspended in SurePath LBC medium) were all reported as negative (100% had *C_T_* values above 38.3 on the HPV16 channel and 34.2 for channels relative to the other eight HPV results) by Onclarity (Table 1).

**TABLE 1:** Mean cycle threshold scores for pooled clinical specimens^*a*^

HPV genotype^*b*^	No. of specimens in group	Mean *C_T_*	Within run		Between runs		Between operators		Between sites		Between reagent lots		Between days		Total	
			SD	%CV	SD	%CV	SD	%CV	SD	%CV	SD	%CV	SD	%CV	SD	%CV
16	159	35.22	1.52	4.3	0	0	0	0	0	0	0.29	0.8	0.32	0.9	1.57	4.5
18	156	30.47	1.08	3.5	0	0	0.08	0.3	0.3	1.0	0	0	0	0	1.11	3.6
45	156	33.35	1.78	5.3	0.34	1.0	0	0	0.25	0.8	0	0	0	0	1.83	5.5
31	156	33.21	1.81	5.4	0	0	0	0	0	0	0.51	1.5	0	0	1.86	5.6
33/58	156	30.73	1.38	4.5	0.20	0.7	0	0	0.12	0.4	0.19	0.6	0	0	1.41	4.6
52	156	30.08	0.79	2.6	0.24	0.8	0	0	0.33	1.1	0	0	0	0	0.87	2.9

a Abbreviations: *C_T_*, cycle threshold; %CV, percent coefficient of variation; HPV, human papillomavirus.

b One hundred percent of HPV-negative specimens (clinical matrix only) were associated with *C_T_* values above the cutoff for a positive result (38.3 on the HPV16 channel and 34.2 for channels relative to the other eight HPV results).

### Onclarity assay results from pre- and postcytology aliquot specimens.

**FIG 2:**
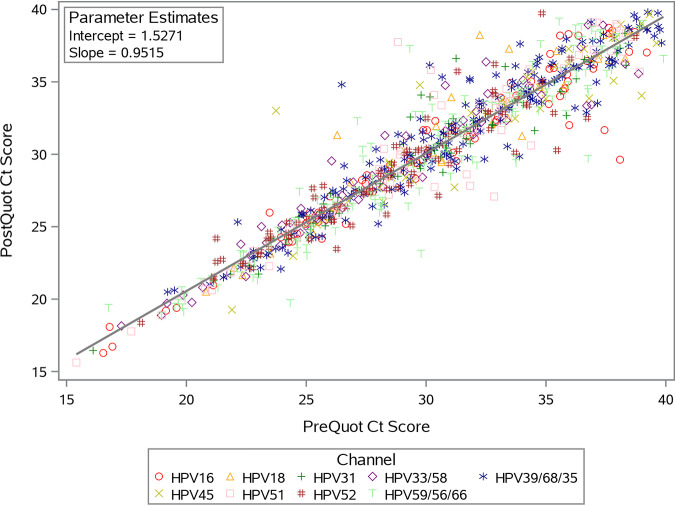
Pre- and postcytology aliquot specimens obtained from the baseline screening phase of the Onclarity trial. The two sample types were tested by the Onclarity assay, and the results (either HPV16, -18, -31, -33/58, -45, -51, -52, -35/39/68, or -56/59/66) are plotted by the mean *C_T_* scores for the precytology aliquot (PreQuot) on the *x* axis and the postcytology aliquot (PostQuot) on the *y* axis. The solid line represents the regression line for the best fit across the data points.

Individual Onclarity results from precytology aliquot versus postcytology aliquot specimens were compared. The total numbers of individual results were as follows: 77 for HPV16, 34 for HPV18, 65 for HPV31, 59 for HPV33/58, 42 for HPV45, 72 for HPV51, 80 for HPV52, 195 for HPV35/39/66, and 149 for HPV56/59/66. Individual Onclarity assay *C_T_* score results are plotted in Fig. 2, with those from the precytology aliquot on the *x* axis and those from the postcytology aliquot on the *y* axis. Although there was a slight difference in the distribution of *C_T_* scores between pre- and postcytology aliquot groups, the results corresponding to precytology aliquots and postcytology aliquots were linear and represented a one-to-one correlation.

Table 2 shows four categories (ASCUS, >ASCUS, NILM, and any cytology), which correspond to cytology/HPV triage (ASCUS) for women ≥21 years of age, cotesting (NILM) for women ≥30 years of age, and the primary screening population (any cytology) for women ≥25 years of age. Positive and negative percent agreements were high for all cytology categories, and the overall percent agreement between the pre- and postcytology aliquot specimens was >98% for all cytology categories (Table 2). Comparisons of the postcytology aliquot results relative to the precytology aliquot results showed 96.1% (73/76), 100% (83/83), 89.9% (160/178), and 92.2% (353/383) concordance rates for positive results from the ASCUS, >ASCUS, NILM, and any cytology (≥25 years of age) groups, respectively. In addition, concordance rates for the postcytology aliquot specimens compared to the precytology aliquot specimens were 100% (129/129), 90.5% (19/21), 98.9% (2,431/2,457), and 98.9% (3,052/3,087) for negative results from the ASCUS, >ASCUS, NILM, and any cytology (≥25 years of age) groups, respectively. The majority of the discordant results were from women with NILM cytology, and the discordant results also split across the two sample types (precytology aliquot positive/postcytology aliquot negative and precytology aliquot negative/postcytology aliquot positive) (Table S3). Approximately 85% of the discordant results were close to the clinical cutoff of the assay (data not shown).

**TABLE 2:** Percent agreement for Onclarity assay results between pre- and postcytology aliquot specimens^*a*^

Population (age [yrs])	Positive % agreement (95% CI)	Negative % agreement (95% CI)	Overall % agreement (95% CI)
NILM (≥30)	86.0 (80.3, 90.3)	99.3 (98.9, 99.5)	98.3 (97.8, 98.8)
ASCUS (≥21)	100 (95.0, 100)	97.7 (93.5, 99.2)	98.5 (95.8, 99.5)
>ASCUS (≥21)	97.6 (91.8, 99.4)	100 (83.2, 100)	98.1 (93.3, 99.5)
Primary screening (≥25)	91.0 (87.7, 93.4)	99.0 (98.6, 99.3)	98.1 (97.6, 98.5)

a Abbreviations: CI, confidence interval; ASCUS, atypical squamous cells of undetermined significance; NILM, negative for intraepithelial lesions or malignancies.

Mean *C_T_* scores were determined for Onclarity results from specimens that were positive for any high-risk HPV genotype (*n* = 773) or the individual genotype HPV16 (*n* = 77), HPV18 (*n* = 34), or HPV45 (*n* = 42) (Table 3). Pre- and postcytology aliquot mean *C_T_* scores were close across all four test groups, with the mean difference (postcytology aliquot − precytology aliquot) being no greater than 0.31 cycle from zero. Statistical analyses revealed no significant difference between the mean *C_T_* scores from pre- and postcytology aliquot specimens for any of the genotype categories.

**TABLE 3:** Onclarity assay mean cycle threshold scores for pre- and postcytology aliquot specimens^*a*^

HPV genotype	No. of specimens in group	Postcytology mean *C_T_*	Precytology mean *C_T_*	Mean *C_T_* difference (postcytology − precytology)	Lower 95% CI	Upper 95% CI	*P* value
Pooled^*b*^	773	30.31	30.25	0.06	−0.06	0.18	0.3298
16	77	30.19	30.33	−0.14	−0.51	0.22	0.4353
18	34	31.55	31.25	0.31	−0.32	0.94	0.3299
45	42	32.58	32.65	−0.07	−0.75	0.62	0.8462

a Abbreviations: *C_T_*, cycle threshold; CI, confidence interval; HPV, human papillomavirus.

b Mean *C_T_* results for the total study population (≥21 years of age) for all nine, combined HPV genotype channels (three channels for each of three wells). One hundred percent of HPV-negative specimens (clinical matrix only) were associated with *C_T_* values above the cutoff for a positive result (38.3 on the HPV16 channel and 34.2 for channels relative to the other eight HPV results).

### Onclarity results based on specimen collection device.

**FIG 3:**
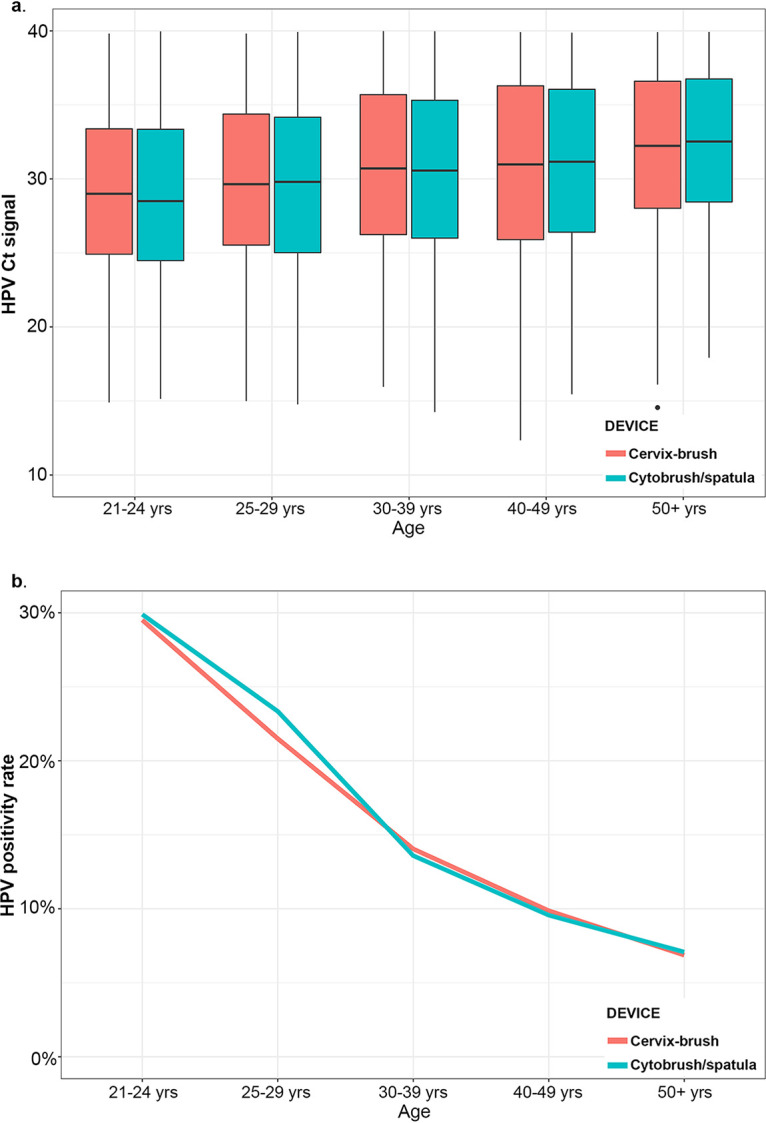
Specimens were obtained from participants of the Onclarity trial with either the Cervex-Brush or the Cytobrush (or Cytobrush/spatula). (a) Results (mean *C_T_* scores) plotted for both types of collection devices and stratified by age group. (b) Positivity rates from the specimens in panel a for each collection device and stratified by age group.

Onclarity performances were compared following collection with either the Cervex-Brush or the Cytobrush (or Cytobrush/spatula) in three screening populations: ASCUS, ≥21 years of age (*n* = 989 for Cervex-Brush and *n* = 964 for Cytobrush); NILM, ≥30 years of age (*n* = 11,145 for Cervex-Brush and *n* = 11,139 for Cytobrush); and primary screening, ≥25 years of age (*n* = 14,858 for Cervex-Brush and *n* = 14,654 for Cytobrush). To compare Onclarity performances for each collection device, the *C_T_* values were averaged for all samples with a signal (*C_T_* < 40) on the Viper LT system (*n* = 427 and *n* = 424 for Cervex-Brush and Cytobrush, respectively, in the ASCUS population; *n* = 1,427 and *n* = 1,390 for Cervex-Brush and Cytobrush, respectively, in the NILM population; and *n* = 2,637 and *n* = 2,586 for Cervex-Brush and Cytobrush, respectively, in the primary screening population) (Table S4). No significant difference was observed between the Cervex-Brush and the Cytobrush (or Cytobrush/spatula) across the three screening populations or by age group (Fig. 3a and Table S4). In addition, no significant difference was observed between the Cervex-Brush and Cytobrush (or Cytobrush/spatula) *C_T_* scores related to the detection of the internal control (HBB gene) (Table S4 and Fig. S3). The HPV positivity rates (for those samples with signals of ≤38.3 for the HPV16 channel and ≤34.2 for the other eight HPV channels) for specimens collected with the Cervex-Brush and Cytobrush (or Cytobrush/spatula) devices were not significantly different across all three screening populations. In addition, positivity rates with both collection devices tended to decrease with increasing age in the primary screening population (Fig. 3b and Table 4). Positivity rates were not significantly different between devices when results were stratified by age (21 to 24, 25 to 29, 30 to 39, 40 to 49, and ≥50 years of age) (Table 4).

**TABLE 4:** Onclarity assay positivity rate following collection by either Cytobrush/spatula or Cervex-Brush^*a*^

Population	Cervex-Brush		Cytobrush/spatula		*P* value by Fisher’s exact test
	No. of positive specimens/total no. of specimens	% positive specimens	No. of positive specimens/total no. of specimens	% positive specimens	
ASCUS (≥21 yrs old)	381/989	38.5	382/964	39.6	0.6428
NILM (≥30 yrs old)	895/11,145	8.0	866/11,139	7.8	0.4869
Primary Screening (≥25 yrs old)	1,888/14,858	12.7	1,860/14,654	12.7	0.9721
Age (yrs)					
21–24	597/2,023	29.5	567/1,897	29.9	0.8066
25–29	603/2,805	21.5	613/2,626	23.3	0.1036
30–39	679/4,834	14.0	631/4,643	13.6	0.5319
40–49	362/3,667	9.9	358/3,741	9.6	0.6663
≥50	244/3,552	6.9	258/3,644	7.1	0.7461

a Abbreviations: ASCUS, atypical squamous cells of undetermined significance; NILM, negative for intraepithelial lesions or malignancies.

## DISCUSSION

The results presented here demonstrate the high reproducibility of Onclarity (within run, between runs, between operators, between sites, between reagent lots, and between days). Onclarity met reproducibility criteria for contrived specimens containing individual genotypes 16, 18, and 45 and for pooled clinical specimens positive for either HPV16, -18, -45, -31, -33/58, or -52. The overall agreement of the results from the Onclarity assay using precytology aliquot and postcytology aliquot samples for each of the subject populations was above 98%, with the lower bound of the 95% confidence interval being ≥93%. Finally, HPV positivity rates and HPV mean *C_T_* scores, both overall and when stratified by age groups, were not statistically different for the two collection devices (Cervex-Brush or Cytobrush [or Cytobrush/spatula]) investigated here (18).

Results from the contrived HPV16 specimens showed a slightly lower percent agreement than the expected result for HPV16 high-negative specimens. As shown in Table S2 in the supplemental material, the majority of the discordance involving the high-negative HPV16 contrived specimens was based on a difference in one location (site 3) and one lot (lot 2). It is not clear that these two instances represent a true depiction of Onclarity assay performance for differentiating HPV16-negative specimens from HPV16-positive specimens around the cutoff. The clinical cutoff *C_T_* value for HPV16 (38.3) is approximately 4 cycles higher than that for the other eight Onclarity results (34.2), which may explain the low reproducibility of results for the HPV16 high-negative specimens relative to the other genotype results. However, the Onclarity assay has been clinically validated for HPV16 detection, and previous results for HPV16 from screening populations have demonstrated good specificity and positive predictive values for the detection of the individual HPV16 genotype (5, 11, 15, 19–24). In addition, the highly reproducible results observed here for the Onclarity assay regarding contrived and pooled clinical specimens are consistent with previous work. Ejegod and colleagues demonstrated high reproducibility with good intralaboratory agreement (98.6%) and kappa value (0.967) and good interlaboratory agreement (98.4%) and kappa value (0.962) for Onclarity assay-positive/negative results from specimens collected in PreservCyt LBC medium in a subset of an English screening population (12). Similarly, Ejegod and colleagues observed good intra- and interlaboratory reproducibility with the Onclarity assay from specimens collected in SurePath LBC medium from a Danish population (13). In this study, the greatest variation for pooled clinical specimen results was observed within Onclarity assay runs. This was not unexpected as LBC specimens are inherently nonhomogeneous. They are composed of sheaths of sloughed-off, exfoliated cells that are stored in a fixative, which can lead to clumping. For viral signals, this is further exacerbated by the focal nature of HPV infections, often representing just a small fraction of the total cell population in a specimen. All other factors, including between runs, between operators, between sites, between reagent lots, and between days, showed relatively low variation in results compared to the within-run results.

The Onclarity assay is an FDA-approved and CE-marked HPV test for which clinical validation has previously been established (5, 11–15, 17, 20–32). In accordance with the criteria of Meijer et al. (6) for a clinically validated HPV assay, the Onclarity assay has been shown to be noninferior to Hybrid Capture-II (HC2) HPV assay (Qiagen, Germantown, MD) (≥90% of HC2 sensitivity and ≥98% of HC2 specificity) (11, 12). In addition, since 2011, the Onclarity assay has been compared with and validated against other established HPV assays in large screening/opportunistic screening population studies (12, 13, 15, 21, 28, 30, 31), clinical comparison studies (11–13, 20), studies involving referral populations (20, 22–24, 27, 29, 32, 33), and studies involving large repositories of well-characterized specimens (5, 26) derived from populations from numerous countries, including the United States, Belgium, Denmark, Italy, England, Japan, and Mexico. The clinical and analytical performance of the Onclarity assay for extended genotyping has been previously established in studies using comparators, including sequencing-based assays (5, 20, 24, 29, 31, 32, 34).

The Onclarity assay is the only HPV assay approved for extended genotyping (reporting of individual genotypes 16, 18, 31, 45, 51, and 52 and reporting of grouped results for genotypes 33/58, 35/39/68, and 56/59/66) in the primary screening population (≥25 years of age). Importantly, the Onclarity assay provides coverage of the three HPV genotypes (HPV16, -18, and -45) that represent approximately 77% of invasive cervical cancer (35) and for adenocarcinoma, which has previously been difficult to detect by cytology-based screening alone (36). Wright et al. demonstrated comparable sensitivities and specificities for Onclarity and HC2 for cervical intraepithelial neoplasia grade 2 or higher (≥CIN2) and ≥CIN3 in the ASCUS triage population (23). In addition, Stoler et al. demonstrated comparable sensitivities and specificities for Onclarity and HC2 for ≥CIN2 and ≥CIN3 in the cotesting population (15). Here, we observed a high concordance for HPV16, -18, and -45 with the expected result for contrived specimens corresponding to low- and medium-positive and high-negative concentrations.

In addition to the >95% agreement with the expected results for HPV16-, HPV18-, and HPV45-positive contrived specimens, the mean *C_T_* scores of individual results for HPV16, -18, -45, -31, -33/58, and -52 from HPV-positive pooled clinical specimens also demonstrate the reproducibility of the Onclarity assay. In addition, 100% of HPV-negative specimens (clinical matrix only) were associated with *C_T_* values above the cutoff for a positive result (38.3 on the HPV16 channel and 34.2 for channels relative to the other eight HPV channels). This reproducibility is important as countries in North America, Europe, Australia, and Asia continue to consider extended and full genotyping as a triage approach to improve risk detection for high-grade cervical disease during HPV primary screening. Publications from both the Onclarity clinical trial and Kaiser Permanente Northern California have previously demonstrated the potential benefit of extended genotyping to identify either those with NILM cytology as being at high-enough risk for a referral for colposcopy (e.g., those with NILM cytology and positive for HPV16 or -31) or those with ASCUS/low-grade squamous intraepithelial lesion (LSIL) as being of low-enough risk to return for follow-up as opposed to a referral for immediate colposcopy (e.g., those with ASCUS/LSIL cytology and positive for HPV56) (21, 22, 32). A recent systematic review outlines further evidence for extended/full genotyping as an effective means for triage in both U.S.-based populations and populations outside the United States (37).

As many HPV assays are nucleic acid amplification based, precytology aliquot specimens are typically preferred for HPV testing prior to LBC processing. However, current cervical cancer screening recommendations, both inside and outside the United States, vary based on the age of the screening population (among other factors, including prior screening/treatment status). Approaches to cervical cancer screening also vary from country to country. In Europe, for example, approximately 55% of countries utilize cytology as the primary screening modality, with 45% of countries utilizing some combination of cytology and HPV testing (9). Depending on the country or region, specimens for HPV testing could be aliquoted either before or after the specimen is processed for cytology. Therefore, it is important to understand how precytology aliquot and postcytology aliquot specimens may or may not vary for HPV assay performance. The overall agreement of the results from the Onclarity assay using precytology aliquot and postcytology aliquot specimens, representing three cervical cancer screening populations, was high. However, the discordant results that were observed are not unexpected, especially in samples close to the cutoff of the Onclarity assay. As discussed above, LBC specimens are inherently nonhomogeneous, which warrants confirmation of pre- and postcytology analyses to confirm within-specimen consistency. Although specimens are not routinely tested twice, laboratories may test specimens either before or after cytology, depending on their preferred workflow and standard-of-care screening paradigm (e.g., cotesting versus HPV primary screening). In addition, a lower agreement was observed for the pre- and postcytology results in the NILM cytology group. NILM cytology positive for HPV *a priori* represents early or receding infections; thus, enrichment is likely occurring in this cytology group for HPV-positive results that are close to the clinical cutoff of the assay. Qualitative HPV assays show more variability at low infection levels.

Here, the collection device had no overall impact on the HPV result. In addition, there was no observed effect of the collection device type across age groups, and therefore, either collection device should be effective for different screening populations (ASCUS triage, ≥21 years; cotesting, ≥30 years; and ≥25 years). The squamocolumnar junction (SCJ) is an anatomical area in which cellular transformation occurs at a high rate and is a common region in which abnormal cells develop. With age, the cervical transformation zone (including its distal edge, the SCJ) recedes into the cervical canal (27), which renders LBC collection from the SCJ challenging. Here, the choice of collection device did not impact the ability to detect HPV for the ≥40- and ≥50-year age groups.

### Limitations.

Clinical specimens used in this study were obtained from the Onclarity trial, a large cervical cancer screening trial conducted in the United States, which has been described previously. Therefore, some aspects of bias or imprecision associated with the experimental design or procedures related to the Onclarity trial may apply to these analyses. These would include some types of partial verification bias when stratifying results by age or cytology result. This was addressed for results from the Onclarity trial previously through a statistical methodology to adjust for verification bias, which was not conducted during stratification by cytology results for pre- and postcytology and collection device analyses. In addition, classification bias due to a lack of a true reference for pre- and postcytology and collection device analyses on HPV detection may have led to some inaccuracies in our results here. Some form of analytic bias could have occurred here, especially between study sites, which has not been explained and may have impacted our results (for example, results for the high-negative HPV16 specimens). Finally, as discussed above, regarding the HPV16 high-negative results, bias could have affected the accuracy of our results for HPV16 compared to the other eight HPV results as the difference in the HPV signal-negative (38.3 < *C_T_* < 40) and signal-positive (*C_T_* ≤ 38.3) *C_T_* values is smaller (the cutoff is closer than the limit of detection) than that for the other eight HPV results: signal negative, 34.2 < *C_T_* < 40; signal positive, *C_T_* ≤ 34.2. Finally, histological outcomes were not used here to determine whether Onclarity assay results involving clinical specimens corresponded to performance compared to histological outcomes. However, the objective of this study was to determine the analytical performance of the Onclarity assay using clinical and contrived specimens, irrespective of the ability of the Onclarity assay to detect cancer or precancer. The clinical performance of the Onclarity assay, compared to histology as a reference, has been described extensively elsewhere (11, 15, 20, 23).

### Conclusion.

Overall, the results here characterize the impact of preanalytical activities on Onclarity assay reproducibility and provide evidence for the potential flexibility of the Onclarity assay within different workflows during cervical cancer screening. This includes sample collection devices, aliquoting order, and other laboratory workflow practices. Regardless of each of these factors, the results obtained with the Onclarity assay on the Viper-LT system were robust and reproducible.

## Supplementary Material

Supplemental file 1
