# Supplementary material for: Analytical and Clinical Sample Performance Characteristics of the Onclarity Assay for the Detection of Human Papillomavirus
Source: J Clin Microbiol. 2020 Dec 17;59(1):e02048-20. doi: 10.1128/JCM.02048-20 (PMC7771472; doi:10.1128/JCM.02048-20)
Supplement: Supplemental file 1 [file JCM.02048-20-s0001.pdf]

SUPPLEMENTAL MATERIAL
TABLE S1

Table S1. Acceptance criteria for establishment of Onclarity performance reproducibility

Preparation	Genotype	Concentration	Acceptance criteria
**Contrived**	HPV16, 18, or 45	High negative	94% HPV negative
	HPV16, 18, or 45	Low positive	94% HPV positive
	HPV16, 18, or 45	Moderate positive	98% HPV positive
**Pooled clinical**	HPV16	Moderate positive	34.2≤ mean Ct ≤38.3
	HPV18, 45, 31, 33/58, or 52	Moderate positive	29.6≤ mean Ct ≤34.2
	HPV negative	n/a	98% HPV negative
**Any**[a]	HBB internal controls	n/a	Ct ≤34.2 for all replicates

Abbreviations: HPV, human papillomavirus; C_5;concentration at which specimens are expected to be negative, 95% of the time and positive, 5% of the time; C_{95}, concentration at which specimens are expected to be negative, 5% of the time and positive, 95% of the time; $3XC_{95}$, concentration at which specimens are expected to be positive 100% of the time; Ct, PCR cycle threshold; n/a, not applicable; HBB, human beta globin gene

[a]Applies to both contrived and pooled clinical specimens

TABLE S2

Table S2.

	Negative						Low						Moderate					
	HPV 16		HPV 18		HPV 45		HPV 16		HPV 18		HPV 45		HPV 16		HPV 18		HPV 45	
**Testing site**	**n**	**% Negative**	**n**	**% Negative**	**n**	**% Negative**	**n**	**% Positive**	**n**	**% Positive**	**n**	**% Positive**	**n**	**% Positive**	**n**	**% Positive**	**n**	**% Positive**
**Site 1**	54	92.6	54	100	54	100	54	100	54	100	54	94.4	53	100	54	100	54	100
**Site 2**	54	98.1	51	100	51	100	54	98.1	51	96.1	51	94.1	54	100	51	100	51	98.0
**Site 3**	51	80.4	51	100	51	100	51	100	51	98.0	51	98.0	51	100	51	100	51	100

	Negative						Low						Moderate					
	HPV 16		HPV 18		HPV 45		HPV 16		HPV 18		HPV 45		HPV 16		HPV 18		HPV 45	
**Reagent lot**	**n**	**% Negative**	**n**	**% Positive**	**n**	**% Negative**	**n**	**% Positive**	**n**	**% Positive**	**n**	**% Positive**	**n**	**% Positive**	**n**	**% Positive**	**n**	**% Positive**
**Lot 1**	51	96.1	54	100	54	100	51	100	54	96.3	54	96.3	50	100	54	100	54	98.1
**Lot 2**	54	83.3	51	100	51	100	54	98.1	51	100	51	96.1	54	100	51	100	51	100
**Lot 3**	54	92.6	51	100	51	100	54	100	51	98.0	51	94.1	54	100	51	100	51	100

	Negative						Low						Moderate					
	HPV 16		HPV 18		HPV 45		HPV 16		HPV 18		HPV 45		HPV 16		HPV 18		HPV 45	
	**n/N**	**% Positive (95% CI)**	**n/N**	**% Positive (95% CI)**	**n/N**	**% Positive (95% CI)**	**n/N**	**% Positive (95% CI)**	**n/N**	**% Positive (95% CI)**	**n/N**	**% Positive (95% CI)**	**n/N**	**% Positive (95% CI)**	**n/N**	**% Positive (95% CI)**	**n/N**	**% Positive (95% CI)e**
**All data Combined**	144/ 159	91.6 (85.0, 94.2)	156/ 156	100 (97.6, 100)	156/ 156	100 (97.6, 100)	158/ 159	99.4 (96.5, 99.9)	153/ 156	98.1 (94.5, 99.3)	149/ 156	95.5 (91.0, 97.8)	158/ 158	100 (97.6, 100)	156/ 156	100 (97.6, 100)	155/ 156	99.4 (96.5, 99.9)

Abbreviations: HPV, human papillomavirus

TABLE S3

Table S3. Comparison of Onclarity assay pre-cytology aliquot and post-cytology aliquot results

Onclarity—Post-cytology aliquot result	Onclarity—Pre-cytology aliquot result							
	**ASCUS** (≥21 years)		**>ASCUS** (≥21 years)		**NILM** (≥30 years)		**All Subjects** (≥25 years)	
	**Positive**	**Negative**	**Positive**	**Negative**	**Positive**	**Negative**	**Positive**	**Negative**
**Positive**	73	3	83	0	160	18	353	30
**Negative**	0	129	2	19	26	2,431	35	3,052
**Total**	73	132	85	19	186	2,449	388	3,082

Abbreviations: ASCUS, atypical squamous cells-undetermined significance; NILM, negative for intraepithelial lesions or malignancies

TABLE S4

Table S4. Onclarity assay—mean cycle threshold scores for the HBB (internal control) gene and pooled HPV genotype results

**Human papillomavirus** (pooled genotypes)						
	**Cytobrush/spatula**		**Cervex-brush**			**Two-sample t-test**
**Population**	**Group number**	**Mean Ct** (SD)	**Group number**	**Mean Ct** (SD)	**Difference (95%CI)**	**p-value**
**ASCUS** (≥21 years)	424	27.23 (5.12)	427	27.29 (5.34)	-0.06 (-0.77, 0.64)	0.8636
**NILM** (≥30 years)	1,390	32.44 (4.95)	1,427	32.37 (5.02)	0.07 (-0.30, 0.44)	0.7035
**Primary Screening** (≥25 years)	2,586	30.54 (5.73)	2,637	30.50 (5.79)	0.03 (-0.28, 0.35)	0.8272
**Age 21-24 years**	706	28.87 (5.63)	744	29.06 (5.51)	-0.19 (-0.76, 0.39)	0.5203
**Age 25-29 years**	784	29.57 (5.66)	784	29.63 (5.66)	-0.06 (-0.62, 0.50)	0.8387
**Age 30-39 years**	870	30.42 (5.74)	936	30.55 (5.77)	-0.13 (-0.66, 0.40)	0.6403
**Age 40-49 years**	516	30.93 (5.83)	539	30.73 (6.01)	0.19 (-0.52, 0.91)	0.5897
**Age ≥50 years**	416	32.09 (5.32)	378	31.84 (5.51)	0.25 (-0.51, 1.00)	0.5244

**Human β-globin**						
	**Cytobrush/spatula**		**Cervex-brush**		**Two-sample t-test**	
**Population**	**Group number**	**Mean Ct** (SD)	**Group number**	**Mean Ct** (SD)	**Difference (95%CI)**	**p-value**
**ASCUS** (≥21 years)	964	24.36 (1.63)	989	24.65 (1.88)	-0.29 (-0.45, -0.14)	0.0002
**NILM** (≥30 years)	11,139	24.68 (1.73)	11,145	24.87 (1.95)	-0.19 (-0.24, -0.14)	<.0001
**Primary Screening** (≥25 years)	14,654	24.62 (1.73)	14,,858	24.83 (1.95)	-0.21 (-0.25, -0.17)	<.0001
**Age 21-24 years**	1,897	24.37 (1.65)	2,023	24.65 (1.94)	-0.27 (-0.38, -0.16)	<.0001
**Age 25-29 years**	2,626	24.42 (1.73)	2,805	24.73 (1.97)	-0.30 (-0.40, -0.20)	<.0001
**Age 30-39 years**	4,643	24.43 (1.78)	4,834	24.74 (2.03)	-0.31 (-0.39, -0.23)	<.0001
**Age 40-49 years**	3,741	24.68 (1.81)	3,667	24.92 (2.00)	-0.24 (-0.33, -0.15)	<.0001
**Age ≥50 years**	3,644	24.94 (1.50)	3,552	24.93 (1.75)	0.01 (-0.06, 0.09)	0.7720

Abbreviations: ASCUS, atypical squamous cells-undetermined significance; NILM, negative for intraepithelial lesions or malignancies

FIGURE S1

Figure S1

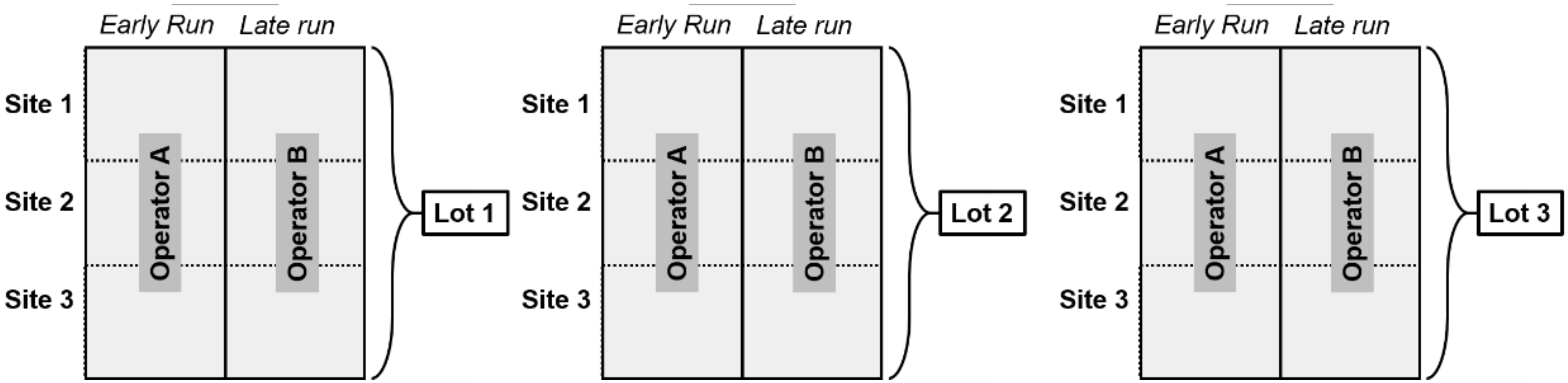

Figure S1. Work flow outline delineating the distribution sample testing by site, lot, and operator.

FIGURE S2

Figure S2

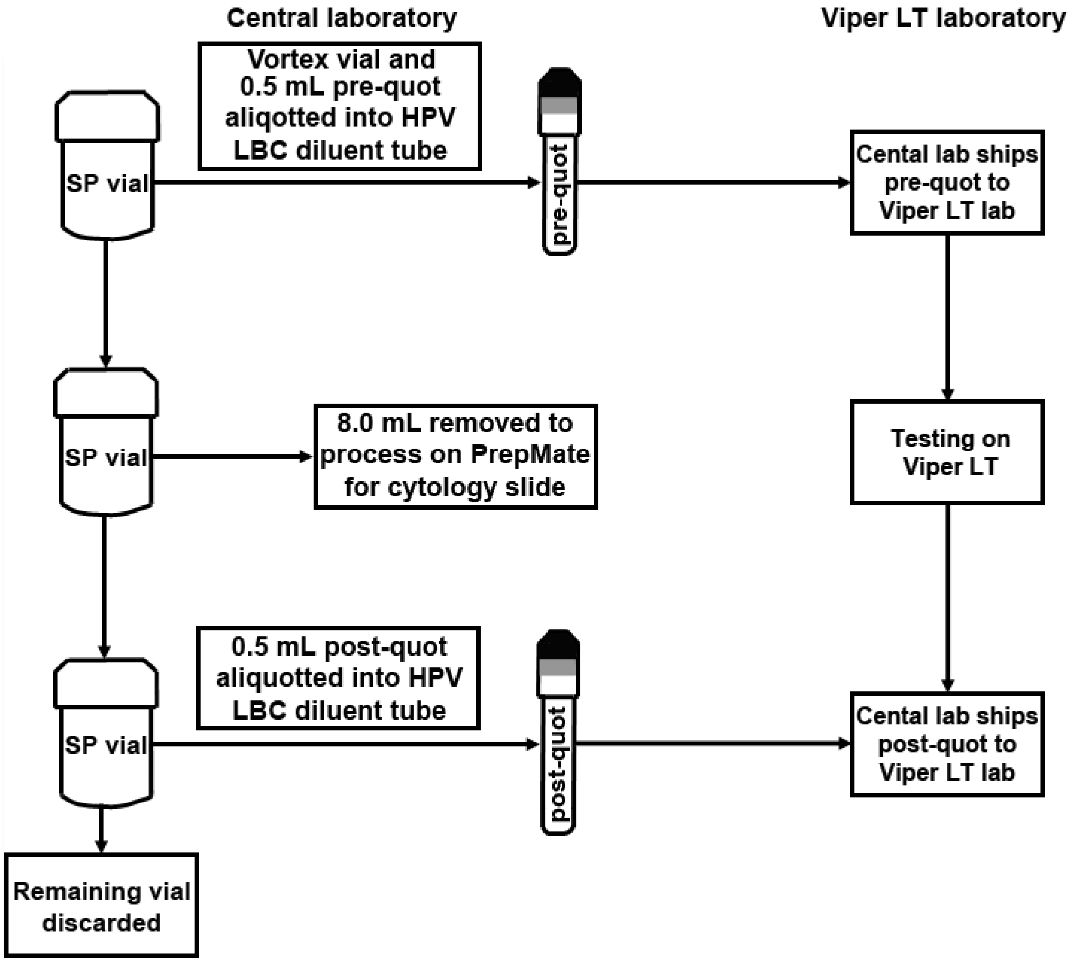

Figure S2. Methodology for aliquot (pre- and post-cytology) acquisition used for HPV testing.

FIGURE S3

Figure S3

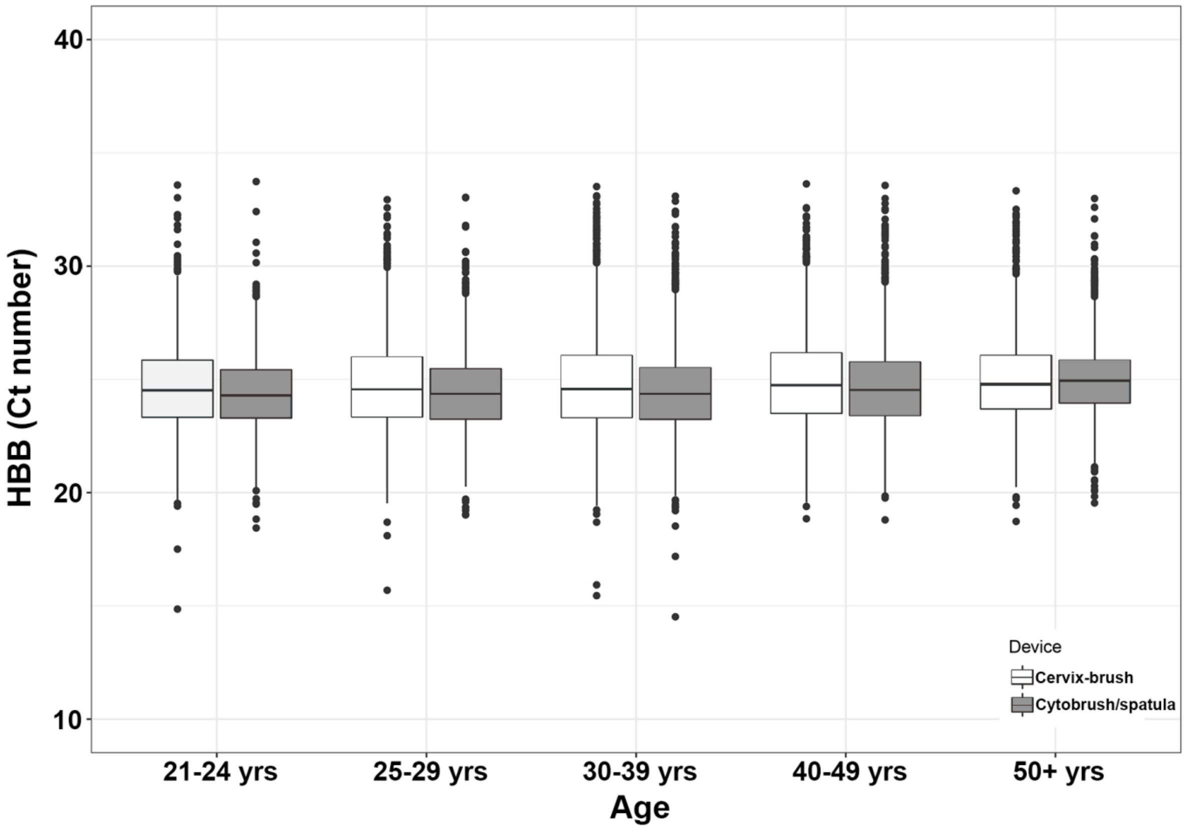

Figure S3. Specimens were obtained from participants of the Onclarity trial with either the Cervex-brush or the Cytobrush (or Cytobrush/spatula). Results for HBB result (mean Ct score) are plotted for both types of collection devices and are stratified by age group.
